# Cost-Sensitive Radial Basis Function Neural Network Classifier for Software Defect Prediction

**DOI:** 10.1155/2016/2401496

**Published:** 2016-09-21

**Authors:** P. Kumudha, R. Venkatesan

**Affiliations:** ^1^Department of Computer Science and Engineering, Coimbatore Institute of Technology, Coimbatore, Tamil Nadu 641 014, India; ^2^Department of Computer Science and Engineering, PSG College of Technology, Coimbatore, Tamil Nadu 641 004, India

## Abstract

Effective prediction of software modules, those that are prone to defects, will enable software developers to achieve efficient allocation of resources and to concentrate on quality assurance activities. The process of software development life cycle basically includes design, analysis, implementation, testing, and release phases. Generally, software testing is a critical task in the software development process wherein it is to save time and budget by detecting defects at the earliest and deliver a product without defects to the customers. This testing phase should be carefully operated in an effective manner to release a defect-free (bug-free) software product to the customers. In order to improve the software testing process, fault prediction methods identify the software parts that are more noted to be defect-prone. This paper proposes a prediction approach based on conventional radial basis function neural network (RBFNN) and the novel adaptive dimensional biogeography based optimization (ADBBO) model. The developed ADBBO based RBFNN model is tested with five publicly available datasets from the NASA data program repository. The computed results prove the effectiveness of the proposed ADBBO-RBFNN classifier approach with respect to the considered metrics in comparison with that of the early predictors available in the literature for the same datasets.

## 1. Introduction

Software fault prediction is always a complex area of research, and software practitioners and researchers have carried out numerous methods to predict where the fault is likely to occur in the software module and their varying degrees of success. These prediction studies result in fault prediction models, which allows software personnel to concentrate on the defect-free software code, thereby resulting in software quality improvement and employing better utility of the resources. The international standard for evaluating the software quality is ISO/IEC 9126. Based on this ISO/IEC 9126 standard, the characteristics of software quality are with respect to internal and external metrics. The key characteristics include efficiency, usability, reliability, maintainability, functionality, and portability. Internal metrics focus only on the product itself without considering its behavior, whereas external metrics focus on the behavior of the product. When software quality comes into picture, then software defect prediction (SDP) plays a major role. Software is described to be of high quality when it is defect-free. This research work mainly concentrates on the internal metrics of the system which include the source code of software systems and not their functions or behavior of the system [1].

**Figure 1:**
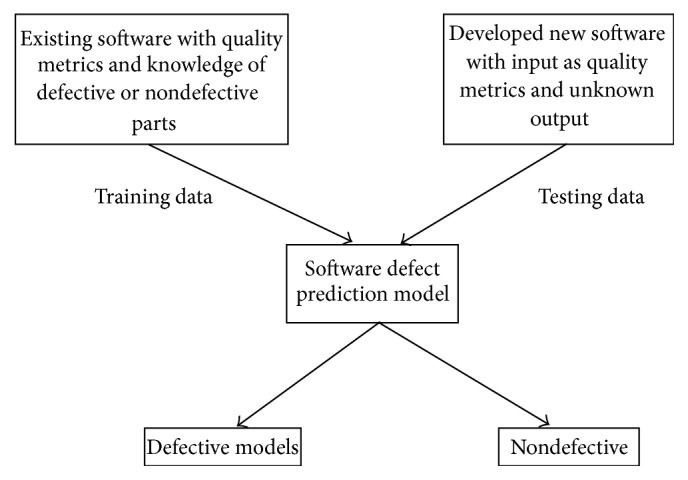
Basic software defect prediction model.

It is to be noted that, for the past two decades, several researchers focused on developing fault-prone software as well as identifying methodologies to detect the software affected by various types of defects [2–4]. The prediction models developed by the researchers perform automatically for software defect prediction before carrying out the manual evaluation process. The developed predicted models should be more effective than the nonpredicted models.  Figure 1 shows the fundamental block diagram of the basic software defect prediction model.

In this research, cost-sensitive neural network model is developed for carrying out the prediction operation. Generally, in numerous cases, the misclassification cost of the majority class is noted to be the least in comparison with that of the minority class. In cases, if a defective model is identified as a nondefective model, then this will lead to higher fixing costs because that software will be employed into the field for utilization [5]. Also, if a nondefective model is identified as a defective model, this will result in unwanted testing carried out on the software, leading to time cost and an increase in testing cost. But this case is quite more acceptable than the previous case, leading to identification of defective model to be nondefective. Hence, this research focuses on developing cost-sensitive neural classifiers considering the two above said cases minimizing the total misclassification costs. The contribution made in this paper includes the development of radial basis function neural network tuned by the proposed adaptive dimension biogeography based optimization and introducing the cost-sensitive measures into the proposed classifier by evolving an objective function. The applicability of radial basis function neural network for various applications is discussed in the following paragraph.

Yang [6] developed radial basis function neural network for discriminant analysis. This work focused on the exploitation of the weight structure of radial basis function neural networks using the Bayesian method. It is expected that the performance of a radial basis function neural network with a well-explored weight structure can be improved. Ghosh-Dastidar et al. [7] developed a novel principal component analysis- (PCA-) enhanced cosine radial basis function neural network classifier. In the first stage, PCA is employed for feature enhancement. The rearrangement of the input space along the principal components of the data improves the classification accuracy of the cosine radial basis function neural network (RBFNN) employed in the second stage significantly. The classification accuracy and robustness of the classifier are validated by extensive parametric and sensitivity analysis.

Lian [8] developed a self-organizing fuzzy radial basis function neural network controller (SFRBNC) to control robotic systems. The SFRBNC uses a radial basis function neural network (RBFN) to regulate the parameters of a self-organizing fuzzy controller (SOFC) to appropriate values in real time. Rubio-Solis and Panoutsos [9] developed an interval type 2 radial basis function neural network (IT2-RBF-NN) incorporating interval type 2 fuzzy sets within the radial basis function layer of the neural network in order to account for linguistic uncertainty in the system's variables.

Jianping et al. [10] modeled a complex radial basis function neural network that is proposed for equalization of quadrature amplitude modulation (QAM) signals in communication channels. The network utilizes a sequential learning algorithm referred to as complex minimal resource allocation network (CMRAN) and is an extension of the M-RAN algorithm originally developed for online learning in real-valued radial basis function (RBF) networks. Lei and Lu [11] proposed an online learning adaptive radial basis function neural network (RBFNN) to deal with measurement errors and environment disturbances to improve control performance. Since the weight matrix of the adaptive neural network can be updated online by the state error information, the adaptive neural network can be constructed directly without prior training.

Kumar et al. [15] developed a novel approach for odor discrimination of alcohols and alcoholic beverages using published data obtained from the responses of thick film tin oxide sensor array fabricated at our laboratory and employing a combination of transformed cluster analysis and radial basis function neural network. The performance of the new classifier was compared with others based on backpropagation (BP) algorithm. Yeung et al. [16] employed support vector machine (SVM), radial basis function neural network (RBFNN), and multilayer perceptron neural network (MLPNN) for solving problems and treating unseen samples near the training samples to be more important.

Karayiannis and Xiong [17] introduced a learning algorithm that can be used for training reformulated radial basis function neural networks (RBFNNs) capable of identifying uncertainty in data classification. This learning algorithm trains a special class of reformulated RBFNNs, known as cosine RBFNNs, by updating selected adjustable parameters to minimize the class-conditional variances at the outputs of their radial basis functions (RBFs). Qiu et al. [18] proposed a Gaussian radial basis function neural network (RBFNN) that was used to preprocess raw EP signals before serving as the reference input. Since the RBFNN has built-in nonlinear activation functions that enable it to closely fit any function mapping, the output of RBFNN can effectively track the signal variations of evoked potentials.

Xie and Leung [19] proposed a novel blind equalization approach based on radial basis function (RBF) neural networks. By exploiting the short-term predictability of the system input, a RBF neural net is used to predict the inverse filter output.

Jafarnejadsani et al. [20] developed an adaptive control based on radial basis function neural network (NN) for different operation modes of variable-speed variable-pitch wind turbines including torque control at speeds lower than rated wind speeds, pitch control at higher wind speeds, and smooth transition between these two modes.

Leung et al. [21] solved the problem of optimum prediction of noisy chaotic time series using a basis function neural network, in particular the radial basis function (RBF) network. Meng et al. [22] modeled a reliable price prediction model based on an advanced self-adaptive radial basis function (RBF) neural network. The proposed RBF neural network model is trained by fuzzy c-means and differential evolution is used to autoconfigure the structure of networks and obtain the model parameters.

Gao et al. [23] developed an approach for seam tracking during high-power fiber laser butt-joint welding. Kalman filtering (KF) improved by the radial basis function neural network (RBFNN) of the molten pool images from a high-speed infrared camera is applied to recursively compute the solution to the weld position equations, which are formulated based on an optimal state estimation of the weld parameters in the presence of colored noises. Chang et al. [24] developed an effective procedure based on the radial basis function neural network to detect the harmonic amplitudes of the measured signal.

Yingwei et al. [25] presented a detailed performance analysis of the minimal resource allocation network (M-RAN) learning algorithm; M-RAN is a sequential learning radial basis function neural network which combines the growth criterion of the resource allocating network (RAN) of Platt with a pruning strategy based on the relative contribution of each hidden unit to the overall network output. Dash et al. [26] presented a new approach for the protection of power transmission lines using a minimal radial basis function neural network (MRBFNN). This type of RBF neural network uses a sequential learning procedure to determine the optimum number of neurons in the hidden layer without resorting to trial and error.

Wong et al. [27] applied the radial basis function (RBF) neural network to low-angle radar tracking. Computer simulations show that the RBF network is capable of tracking both stationary and moving targets with high accuracy. Khairnar et al. [28] developed a new approach using a radial basis function network (RBFN) for pulse compression. In the study, networks using 13-element Barker code, 35-element Barker code, and 21-bit optimal sequences have been implemented. In training these networks, the RBFN-based learning algorithm was used. Jain et al. [29] presented an approach based on radial basis function neural network (RBFNN) to rank the contingencies expected to cause steady state bus voltage violations. Euclidean distance-based clustering technique has been employed to select the number of hidden (RBF) units and unit centers for the RBF neural network.

Wong et al. [30] proposed a radial basis function (RBF) neural network with a new incremental learning method based on the regularized orthogonal least square (ROLS) algorithm for face recognition. It is designed to accommodate new information without retraining the initial network. Platt and Matic [31–34] discussed a fairly general adaptation algorithm which augments a standard neural network to increase its recognition accuracy for a specific user. The basis for the algorithm is that the output of a neural network is characteristic of the input, even when the output is incorrect.

The remainder of the paper is organized as follows. The background of the software prediction models is presented in Section 2. The datasets employed in this research paper are given in Section 3.  Section 4 details the metrics adopted for the prediction model. The proposed prediction model with its algorithm is given in Section 5. The results of the proposed model with its analysis are detailed in Section 6 and the conclusions for the research study are presented in Section 7.

## 2. Background on Software Prediction Models

There exist several statistical and machine learning methods to identify defects in the newly developed software modules: a hybrid instance selection using nearest neighbor [35], distance-based multiobjective particle swarm optimization [36], cost-sensitive boosting neural networks [37], and fuzzy linear regression model [38]. A fuzzy logic based phase wise defect prediction model was validated for twenty pieces of real software project data [39].

Apart from these above said methods, several other prediction models were developed and applied for the open source NASA datasets available at the PROMISE repository [1, 13, 40–52]. Han and Jing [53] employed a high computational wrapper model with a significant improvement in recall rate and* F*-measure. In a similar way, ensemble decision trees and CART were also employed for performing cost-sensitive classification for SDP [54, 55]. A Bayesian regularization (BR) approach is employed to determine the software faults along with Levenberg-Marquardt algorithm and backpropagation algorithm [56]. A call graph based ranking (CGBR) along with the size and complexity metrics was employed to measure the quality of the software [57]. Tabu Search Fault Localization with path branch and bound procedure on software engineering (TSFL-PBB) was employed to overcome the defect on fault localization [58]. Multistage model for software defect density indicator employing the topmost reliability-relevant metrics and fuzzy inference system (FIS) was proposed by Bahadur and Yadav [59]. Also, simple and multiple linear regression statistical methods have been used for the analysis in detecting defects in software development process [60]. A multiobjetcive defect predictor (MODEP) is developed with a framework on certain multiobjective forms of machine learning techniques like logistic regression and decision trees that are trained using genetic algorithms which lies on cross-project description and local prediction with clusters belonging to similar classes [61].

Data mining approach was employed to show the attributes that predict the defective state of software modules and is used in large software projects to detect defective modules that will cause failures during the software execution process [62]. Meta-analysis of all relevant high quality primary studies of defect prediction was carried out to determine what factors influence predictive performance and as well to predict defect-prone software components [63]. An iterative feature selection approach which repeatedly applies data sampling (to overcome class imbalance) followed by feature selection (to overcome high dimensionality) and finally combines the ranked feature lists from the separate iterations of sampling has been applied to several groups of datasets from two real-world software systems and used two learners to build classification models [64–66]. The predictive ability of the evolutionary computation and hybridized evolutionary computation techniques for defect prediction was applied for datasets from the Apache Software Foundation using the Defect Collection and Reporting System [67]. Zhang et al. [68] analyzed 44 metrics of application level, file level, class level, and function level and made correlation analysis with the number of software defects and defect density; the results show that software metrics have little correlation with the number of software defects but are correlative with defect density.

Software defect prediction model was presented in the early literature for consecutive software products based on entropy and the process starts when the defect is found and ends when the resolution is verified and the defect is closed [69]. Xia et al. [70] proposed an algorithm which combines relief feature selection algorithm and correlation analysis. Support vector machine (SVM) has been developed for software defect prediction using different kernels. Software defect prediction helps improve software quality by building effective predictive classification models using software metrics to facilitate identification of fault-prone modules [71]. Neural network parameter optimization based on genetic algorithm has been developed for software defect prediction and has been applied for datasets from the repositories [72]. A multistage model for software defect density indicator using the topmost reliability-relevant metrics and fuzzy inference system (FIS) has been developed for effective decision support [73]. The ability of requirement metrics for software defect prediction has been carried out employing six machine learning algorithms on the requirement metrics, design metrics, and combination of both metrics [74]. Li et al. [75] applied the concept of fuzzy measure and fuzzy integral to the classification of software defects. A complete description of the summary of software prediction models over various periods of study has been proposed by Han et al. [76]. Random Forest algorithm based software prediction model developing an ensemble classifier was applied for large-scale software system [77].

From the above discussed literature reviews, it is inferred that the early proposed prediction models have not taken into account the misclassification cost of the nondefective and defective modules in large for numerous applications except in few cases [5, 37, 49]. Considering the real-world problems, the rate of misclassification of defective module is more important than the rate of misclassification of nondefective modules. The levels of these misclassifications are defined by their associated cost factors. Thus, there are few efforts made in exploring the associated costs employing neural network architectures employing sampling procedures and threshold levels [37]. The variation is made in the threshold level of the neural network which decides the output until an optimal point is reached with respect to the cost matrix. From [78], it is well noted that the movement of threshold is an appropriate factor to build cost-sensitive neural network architecture.

Radial basis function neural network is an architecture model which employs Gaussian function to enable the network to attain fast convergence. In this work, cost-sensitive RBFNN is developed along with a proposed variant of biogeography based optimization (BBO). BBO is an optimization algorithm developed based on the migration of species from one island to another island [79]. In this research paper, the developed adaptive dimensional biogeography based optimization (ADBBO) is applied to optimize the weights of the proposed cost-sensitive radial basis function neural network (CSRBFNN). The developed approach is validated with the NASA PROMISE repository datasets and is compared with that of the existing traditional and evolutionary algorithms. The computed results prove the effectiveness of the proposed ADBBO based cost-sensitive RBFNN for the considered datasets from the repositories. The cost-sensitive RBFNN is derived based on the fitness function introduced with respect to the software defect prediction problem.

## 3. Description of Datasets [12]

The datasets considered for implementing the proposed approach are the NASA PROMISE repository datasets which are made publicly available for software defect prediction. Tim Menzies is the donor of these public datasets and these datasets include the information on spacecraft instrumentation, satellite flight control, and ground data for storage management. This paper employs the five most widely used datasets from this repository (CM1, JM1, KC1, KC2, and PC1). Each of the considered datasets possesses several software modules with input as the quality metrics. The output of each of the modules includes a defective or nondefective case, which identifies the presence of faults in any of the respective modules. These datasets come from McCabe and Halstead features extractors of the source code developed. These features were defined in the 70s with an idea to objectively characterize code features that are associated with software quality. Both McCabe and Halstead measures are “module” based where a “module” is defined as the smallest unit of functionality. All these five datasets were developed either in C or in C++ language.  Table 1 details the description of the datasets employed in this study.

From Table 1, it can be noted that, for all the considered five datasets, 22 attributes exist including one output attribute which is the goal field (identifies defect or nondefect) and the remaining 21 attributes are the quality metrics acting as input attributes: 5 are the different lines of code measure, 3 are McCabe metrics, 4 are base Halstead measures, 8 are derived Halstead measures, and 1 is a branch count.  Table 2 shows the attribute information of the considered datasets. Instead of using all the 21 attributes in the proposed cost-sensitive RBFNN, out of the specified metrics, feature subselection is carried out and the selected attributes [5] are employed as input to the proposed predictor model.  Table 3 shows the attribute features selected to be used as input for the proposed predictor model. For effective comparison of the proposed approach, the same metrics as in [5] are used as inputs for the proposed software predictor model. It is further noted that the proposed model performs better with the selected attributes as in Table 3, instead of using all the 21 attributes. This results in reducing the computational complexity of the predictor model.

## 4. Metrics Employed for the Prediction Model

Metrics play a major role in developing the predictive model and analyzing the performance of the proposed predictors.  Table 4 represents the confusion matrix based on which the performance of the predictor model is done. The confusion matrix substantiates how the predictor model is classified into various defect categories in comparison with that of their actual classification (observed versus predicted).

The values from the confusion matrix can be combined in order to calculate the various performance measures. The performance measure “Recall” presents the proportion of the correctly predicted defective code, whereas “Precision” specifies the rate of defective prediction or the extent of how far the prediction is originally defective or not. Recall is also called sensitivity, probability of detection (pd), or true positive rate (TPR). Apart from these two measures, there exists an additional measure called probability of false alarm (pf) or false positive rate (FPR) which proposes the proportion of the wrongly classified defective predictions. Based on the above definitions, an optimal predictor should achieve a TPR (pd) of 1, FPR (pf) of 0, and precision of 1. When the computed “pd” and “pf” are plotted, they result in Receiver Operating Characteristics (ROC) curve and from ROC the area under the curve (AUC) is to be noted. AUC is noted to be between 0 and 1, with 1 being the optimal solution point. Certain predictors result in low AUC values but can be tuned further to produce high balance metrics. Prediction accuracy as well plays a major role in validating the efficiency of the proposed model and this describes the proportion of the correctly predicted modules.  Table 5 presents performance measures employed in this research paper for validating the proposed prediction models.

The accuracy is not appropriate for datasets possessing uneven class distribution. The measures as proposed in Table 5 are computed in order to validate the proposed software predictor model.

## 5. The Proposed ADBBO Based Cost-Sensitive RBFNN Predictor Model

Originally, radial basis function neural network is a multilayer feed forward neural network employing Gaussian activation function in place of earlier proposed continuous sigmoidal activation functions [80] in several other neural network models. The advantage of employing radial basis function neural network in this paper is its faster convergence. In order to reduce the time taken for the convergence, the weights of the RBFNN model are optimized employing the proposed adaptive dimensional biogeography based optimization. The RBFNN model along with the optimal weights performs the prediction of defects in the considered datasets to achieve better accuracy with faster convergence. This section details the proposed adaptive dimensional BBO based radial basis function neural network model.

### 5.1. Biogeography Based Optimization: An Overview

The fundamental concepts of how species migrate from one island to another and how new species arise and how species become extinct are the underlying foundation of biogeography [79]. Basically, a habitat is any island or an area which is geographically isolated from other islands. It should be noted that the habitats with a high HSI (Habitat Suitability Index) are noted to have more number of species, whereas those with a low HSI possess a small number of species. Habitats that possess high HSI are noted to have a low species immigration rate as they are nearly saturated with that of the species. Also, the high HSI habitats are noted to possess a high emigration rate. Low HSI habitats tend to have a high species immigration rate due to their sparse populations. Emigration in biogeography based optimization does not infer that the emigrating island loses a feature. The worst solutions in the generated species have the worst features; hence, it possesses a very low emigration rate and a low chance for sharing its features. The species (solution) that have the best features also have the habit of sharing them with the highest probability. This procedure is known as biogeography based optimization.

The concept of emigration and immigration rate is represented by a probabilistic model mathematically. Consider the probability *P*_*S*_ that the habitat contains exactly *S* species at *t*. *P*_*S*_ is noted to change from time *t* to time *t* + Δ*t* as given below:

(1) PSt+Δt=PSt1−λSΔt−μSΔt+PS−1λSΔt+PS+1μS+1Δt,

where *λ*_*S*_ and *μ*_*S*_ represent the immigration and emigration rates of species in the habitat. To have *S* species at time (*t* + Δ*t*), any one of the following conditions is to be met: *S* species were present at time *t*, and there is no occurrence of immigration or emigration between *t* and (*t* + Δ*t*); (*S* − 1) species were present at time *t*; one species immigrated; there were (*S* + 1) species at time *t*; one species emigrated. When the time Δ*t* is noted to be small enough, then the probability of more than one immigration or emigration can be ignored and when Δ*t* → 0 it presents the following equation:

(2) P˙S=−λS+μSPS+μS+1PS+1S=0−λS+μSPS+λS−1PS−1+μS+1PS+11≤S≤Smax−1−λS+μSPS+λS−1PS−1S=Smax.

The equation for emigration rate *e*_*mk*_ and immigration rate *i*_*mk*_ for *k* number of species is given by

(3) emk=Eknimk=I1−kn.

On the value of *E* = *I* and then combining the above said equation, it results in

(4) $$\begin{array}{c} e_{mk}+i_{mk}=E_{k}. \end{array}$$

There exist two main operators in biogeography based optimization: the migration and the mutation. It can be inferred that the mutation rate changes the habitat's Suitability Index Variable (SIV) in a random manner based on the mutation rate. Also, the mutation rate is inversely proportional to the probability of species count. Employing the migration operator, the biogeography based optimization process shares the information among solutions. During the optimal flow, it can be noted that the worst solutions tend to accept more useful and meaningful information from the good solutions. This feature enables the BBO algorithm to be good at exploiting the information based on the current population.

### 5.2. Mathematical Modeling of the Proposed Adaptive Dimensional BBO

The proposed adaptive dimensional biogeography based optimization is built so that it enables the generation of the species based on the earlier species' best solution. ADBBO introduces a parameter called habitat search dimensional rate (*h*_dr_), which is updated online during the habitat search process and is proposed to achieve an acceptable balance between the exploitation (possessing the habitat) and exploration (search for habitat). The habitat search dimensional rate (*h*_dr_) is computed as the ratio of the number of variables disturbed for computing a new solution to that of the total number of variables. This modification is introduced into the traditional biogeography based optimization because even minimal variations in certain variables will result in better candidate solutions and this explores the habitat search space. The habitat search dimensional rate is given by

(5) $$\begin{array}{c} h_{dr}=\frac{T_{d}}{T}, \end{array}$$

where “*T*” is the total number of variables and “*T*_*d*_” is the number of variables disturbed. The ultimate aim of “*h*_dr_” is to tune the exploration search aspects of the traditional BBO algorithm.

At the initial start-up of the process, “*h*_dr_” value is taken to be 0.3 based on several numerical experiments carried out. During the subsequent generation process, the habitat search dimension rate is updated based on the condition of improvement of the solutions in the early generations; that is, if “*n* + 1” iteration is on process, then the checking will be carried out for* n*th generation as given by (6). Therefore,

(6) hdrn+1=hdrnρif improvement exist in best solutionρhdrnif no improvement exists in best solution.

The value of “*ρ*” is fixed at less than 1.0 and this parameter *ρ* is called adaptive dimension parameter and this intends to compute the adaption rate of the forthcoming value of habitat search dimensional rate (*h*_dr_). Higher values of habitat search dimensional rate perform the migration of species through a large number of variables at a time and increase the habitat exploration search process. Lower values of *h*_dr_ increase the exploitation search for occupying the habitats.

This process of ADBBO increases the dimension of the search and the proposed algorithm is aimed at determining new solutions in the enhanced region of the search space. In case when early generation does not show any improvement, the search process will be limited and the algorithm limits itself to that of the existing habitat search space. Thus, in the proposed ADBBO algorithm, the habitat search dimension parameter gets updated at each generation to improve exploration and exploitation search to present a highly efficient optimization process. The maximum and minimum values for habitat search dimension rate are set as 0.5 and 1/*T*, with* T* being the total number of variables in the considered problem. The fixed maximum value will overcome the higher disturbances that might exist in the search space; if these disturbances pertain, they may lead to the slowing down of the convergence of the search process. Also, the set minimum value assures that at least one variable will be chosen by chance and will get updated during the generation of best habitat solution.

Based on the above discussed habitat search dimension rate, for the species solutions which are generated for the best fitness till now in the process, new species will be obtained employing the following:
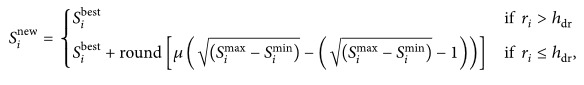
(7)where *S*_*i*_^max^ and *S*_*i*_^min^ stand for the lower and higher ranges of the design variable, respectively, and *S*_*i*_^best^ and *S*_*i*_^new^ are the best values so far carried out during the run and the value of the corresponding variable, respectively. For the respective variable in the generation, “*r*_*i*_” represents uniform random number sampled between 0 and 1. “*μ*” is the random number generated for each of the considered variables based on the standard normal distribution along with a mean zero and standard deviation one.

### 5.3. The Proposed Adaptive Dimensional BBO Algorithm

The proposed algorithm for adaptive dimensional biogeography based optimization is developed by incorporating the above presented adaptive dimensional modeling into the traditional biogeography based optimization process. The adaptive dimensional modeling basically updates the species with respect to the adaptive search dimensional rate (*h*_dr_) and the improvements carried out during the search of best habitat solutions. The BBO process results in the movement of species through the process of habitat search and the position of habitats gets updated during the movement over the search space. This approach of adaptive dimension is introduced before the updating of the position of species and based on the habitat search dimension rate the exploration is carried out and new species are generated and then further fitness for each generated species will be computed and the flow process of BBO is continued. This incorporation of adaptive variation of the species with respect to the dimensional parameter “*h*_dr_” results in faster convergence and improving the exploration of the search space and achieving the near-optimal solution point. Considering the proposed modeling of adaptive dimensional biogeography based optimization in Section 5.2 and converging the proposed model derived with that of the regular BBO, the pseudocode for the proposed ADBBO is as presented in Pseudocode 1.

### 5.4. Radial Basis Function Neural Network Model

**Figure 2:**
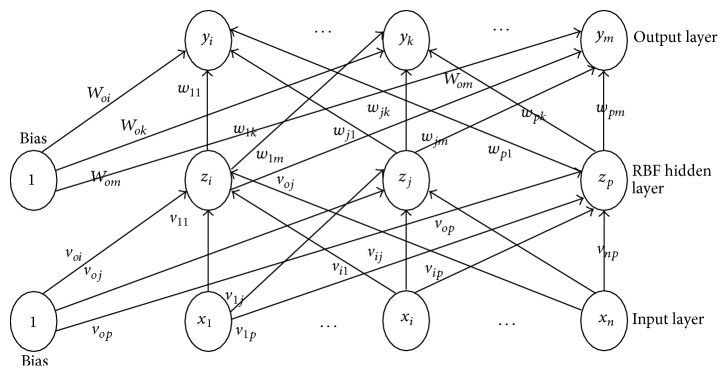
Architecture of radial basis function neural network model.

**Figure 3:**
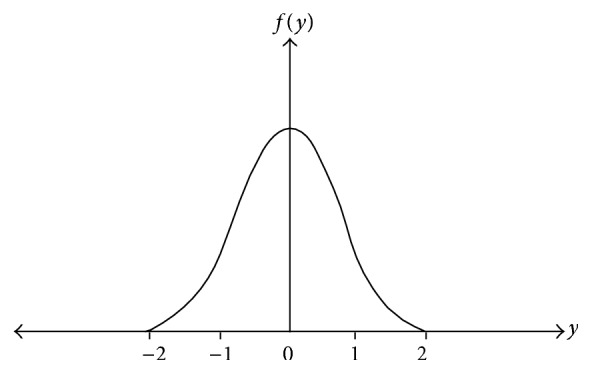
Gaussian activation function in RBFNN model.

Radial basis function neural network performs the training and testing process with a simple gradient descent learning rule and the error obtained during the training process is backpropagated to compute good training efficiency along with the Gaussian distribution function. Radial basis function neural network [81] is a multilayer feed forward neural network with single layer of* z*-hidden units as shown in Figure 2. The* Y* output unit has Wok as bias and* Z*-hidden unit has Vok as bias. The Gaussian activation function employed in RBFNN, which aids the network learning process for faster convergence, is shown in Figure 3.

#### 5.4.1. Learning Algorithm of RBFNN Architectural Model

The learning process of radial basis function neural network consists of the following phases:

- Weight initialization phase.
- Feed forward phase.
- Error radial basis function phase.
- Updating the weights and bias.

The various steps involved in the RBFNN algorithmic flow are as given below.

**Phase 1 (weight initialization phase).**

 

*Step 1*. Initialize the weights between the input layer and hidden layer and between hidden layers and output layer to small random values.

*Step 2*. Initialize the momentum factor and learning rate parameter.

*Step 3*. When the stopping condition is false, perform Steps 4–11.

*Step 4*. For each training dataset vector pair do Steps 5–10.

**Phase 2 (feed forward phase).**

 

*Step 5*. Each input unit belonging to the input layer receives the input signals *x*_*i*_ and transmits these signals to all units in the hidden layer above, that is, to the hidden units.

*Step 6*. Each hidden layer unit (*z*_*j*_, *j* = 1,…, *p*) sums the received weighted input signals. Therefore,

(8) $$\begin{array}{c} z_{-inj}=v_{oj}+\overset{n}{\underset{i=1}{\sum }}x_{i}v_{ij}. \end{array}$$

Applying the continuous Gaussian activation function at this point,

(9) $$\begin{array}{c} Z_{j}=f\left(\;z_{inj}\;\right)\;\text{that is},\;\;f\left(Z_{inj}\right)={e^{-Z_{inj}}}^{2}, \end{array}$$

which sends this signal to all units in the layer above, that is, output units.

*Step 7*. For each of the output units (*y*_*k*_, *k* = 1, …, *m*), compute its net input

(10) $$\begin{array}{c} y_{-inj}=w_{ok}+\overset{p}{\underset{j=1}{\sum }}z_{j}w_{jk} \end{array}$$

and apply Gaussian activation function to the net input for calculating the output signals. Therefore,

(11) $$\begin{array}{c} Y_{k}=f\left(\;y_{-ink}\;\right)\;\text{that is},\;\;f\left(Y_{inj}\right)={e^{-Y_{inj}}}^{2}. \end{array}$$

**Phase 3 (error radial basis function phase).**

 

*Step 9*. Each output unit (*y*_*k*_, *k* = 1,…, *m*) receives a target pattern corresponding to an input pattern; error information term is calculated as follows:

(12) $$\begin{array}{c} \delta_{k}=\left(\;t_{k}-y_{k}\;\right)f^{\prime }\left(\;y_{-ink}\;\right). \end{array}$$

*Step 10*. Each hidden unit (*z*_*j*_,   *j* = 1, …, *n*) sums its delta inputs from units in the layer above as follows:

(13) $$\begin{array}{c} \delta_{-inj}=\overset{m}{\underset{k=1}{\sum }}\delta_{j}w_{jk}. \end{array}$$

Error information term is calculated as follows:

(14) $$\begin{array}{c} \delta_{j}=\delta_{-inj}f^{\prime }\left(\;z_{-inj}\;\right). \end{array}$$

**Phase 4 (updating of weights and bias).**

 

*Step 11*. Compute the weight correction term between the output unit and hidden unit; it is given by the following:

(15) $$\begin{array}{c} \Delta w_{jk}=\alpha \delta_{k}z_{j}+\mu \Delta w_{jk}\left(\;old\;\right). \end{array}$$

And the bias correction term is given by the following:

(16) $$\begin{array}{c} \Delta w_{ok}=\alpha \delta_{k}+\mu \Delta w_{ok}\left(\;old\;\right). \end{array}$$

*Step 12*. Compute the weight correction term between the hidden unit and input unit; it is given by

(17) $$\begin{array}{c} \Delta v_{ij}=\alpha \delta_{j}x_{i}+\mu \Delta v_{ij}\left(\;old\;\right). \end{array}$$

And the bias correction term is given by

(18) $$\begin{array}{c} \Delta v_{oj}=\alpha \delta_{j}+\mu \Delta v_{ok}\left(\;old\;\right). \end{array}$$

*Step 13*. Each output unit (*y*_*k*_, *k* = 1, …, *m*) updates its bias and weights (*j* = 0,…, *p*) and is given by

(19) wjknew=wjkold+Δwjkwoknew=wokold+Δwok.

*Step 14*. Each hidden unit (*z*_*j*_, *j* = 1,…, *p*) updates its bias and weights (*i* = 0, …, *n*) and is given by

(20) vijnew=vijold+Δvijvojnew=vojold+Δvoj.

*Step 15*. Terminate the learning process on reaching the stopping condition. The stopping condition is the number of iterations reached; minimization of the MSE value and the learning rate is decreased to a particular value.

#### 5.4.2. Need of RBFNN Model for Software Defect Prediction Problem

The applicability of Gaussian function enables the radial basis artificial neural network to model nonlinear relationships. The relation between the software quality metrics and their defects is generally complex and is nonlinear in nature. Thus, for handling this complex nonlinearity, a model of artificial neural net RBFNN is a suitable choice for software defect prediction problem. The set goal of the neural net model is to minimize the mean square error (MSE) during the learning process by optimizing the weights of the network (both the input to hidden and hidden to output). The MSE computed is backpropagated in the network and the weights are tuned in a manner to minimize the error. In this paper, error adjustments and tuning for optimal weights are carried out with the proposed adaptive dimensional biogeography based optimization presented in Section 5.3 as well as a new objective function which considers that the cost-sensitivity is taken into account for effective prediction process.

### 5.5. The Proposed ADBBO Cost-Sensitive RBFNN Classifier

This paper proposes a cost-sensitive RBFNN based on the adaptive dimensional BBO for software defect prediction. Originally, RBFNN is a learner that learns based on the weights and bias updating and this basic RBFNN is transformed into a cost-sensitive learner employing a cost error function [5]. The cost parameters considered are the expected cost of misclassification and its normalized value. These cost-sensitive factors are taken based on the false positive error cost and false negative error cost. The objective function of the cost-sensitive RBFNN to be minimized employing the proposed adaptive dimensional BBO is given by the following equation:

(21) minNECM=pf×Pnon-defect-prone+costfalse_negativecostfalse_positive×pfnr×Pdefect-prone,

where “NECM” is the normalized expected cost of misclassification, “pf” is the false positive rate, “pfnr” represents the false negative rate, “cost_false_positive_” is the cost pertaining to false positive error, “cost_false_negative_” is the cost pertaining to false negative error, and “*P*_non-defect-prone_” and “*P*_defect-prone_” are the percentage of non-defect-prone modules and defect-prone modules, respectively.

The pseudocode of the proposed ADBBO-RBFNN is given in Pseudocode 2. During the initial start-up of the learning process, define the variables of the ADBBO algorithm and RBFNN. As the range of values for the software metrics widely varies, a normalization process is required. In this work, min-max (0-1) normalization is employed for the scaling of the considered datasets. The normalization process is carried out individually for training and testing datasets. The training phase is employed to calculate an optimal set of neural network weights, and the performance of the proposed algorithm is then calculated by the determined best optimal weights. RBFNN model initiates its learning process according to the determined optimal weights and calculates the mean square error and the normalized cost of the network. The ratio of Cost_false_negative_ and Cost_false_positive_ (cost ratio) is made based on the expectation from the algorithm. When the cost ratio is higher, Cost_false_negative_ takes a predominant role. On testing process, if the output of the tested network is noted to be higher than 0.5, then the module is fixed to be defect-prone; else, it is categorized as non-defect-prone.

## 6. Experimental Results and Discussion

The proposed adaptive dimensional based biogeography based optimization radial basis function neuronal model is applied for the considered NASA PROMISE repository datasets as described in Section 3. All the considered 5 dataset samples are analyzed employing the cross-validation approach to evaluate the performance of the proposed prediction model. In this paper, a 10-fold cross-validation approach is employed. This procedure randomly splits the datasets into 10 bins of equal size. Hence, for 10 times, 9 bins are selected for training process employing the proposed approach and the remaining 1 bin is used as testing dataset; each time this bin will be a different bin. KC1, KC2, and JM1 datasets were adopted with 10-fold cross-validation and 5-fold cross-validation is used for CM1 and PC1 datasets. The type of cross-validation is chosen based on the defect rate of the datasets under consideration. The optimal parameters chosen for the operation of ADBBO based RBFNN algorithm are tabulated in Table 6.

**Figure 4:**
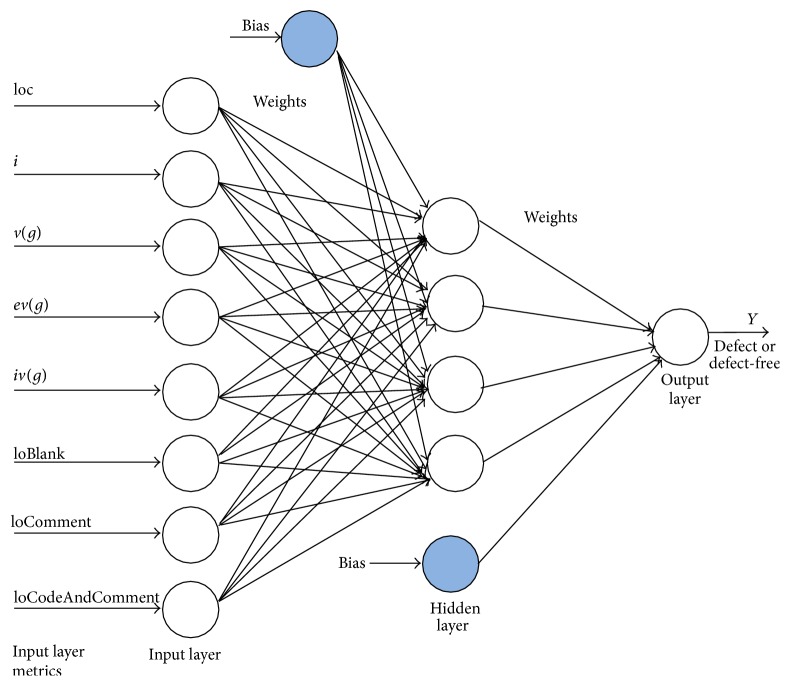
The proposed RBFNN model for JM1 dataset.

The proposed architecture of the RBFNN predictor model sets the number of input neurons equal to that of the attributes selected for each of the datasets as given in Table 3. The main processing in radial basis function neural network is based on the hidden layer neurons and the activation functions between the hidden and output layer neurons. Fixing the number of neurons in the hidden layer is always a complex task in artificial neural network (ANN) modeling and researchers have taken numerous initiatives to fix the number of neurons in the hidden layer [82]. Based on the analysis made in the existing literature for fixation of hidden neurons, in this proposed work, to train the software prediction model for the considered datasets, the number of hidden neurons is set equal to half the number of input neurons so as to reduce the computational complexity. Gaussian function being a nonlinear continuous activation function emulates itself for the faster convergence of the network.  Figure 4 shows the proposed radial basis function neural network model for the JM1 dataset.

In this paper, the proposed adaptive dimensional BBO based radial basis function neuronal classifier is validated for the considered benchmark datasets [12] under two categories to prove its effectiveness: one considering the cost-sensitive part and the other without considering the cost-sensitive part. In each of these cases, the results are compared with various studies from the literature for both non-cost-sensitive prediction and cost-sensitive prediction.

### 6.1. Simulation Results for the Proposed Non-Cost-Sensitive Prediction Model

The costs of false positive rate and false negative rate are not considered in this section during the training process. As a result, (21) which acts as an objective function for ADBBO algorithm to tune for the optimal weights of RBFNN predictor becomes modified as follows:

(22) $$\begin{array}{c} \underset{NECM}{min}=pf\times P_{\text{non-defect-prone}}+pfnr\times P_{\text{defect-prone}}; \end{array}$$

that is, the costs of false positive and false negative are assumed to be of equal weight and, thus, cost_false_negative_/cost_false_positive_ = 1. The simulation results are obtained without considering the cost-sensitive component. The methodology is implemented for NASA PROMISE datasets given in Table 1. The performance results of these datasets are given in Table 7.

**Figure 5:**
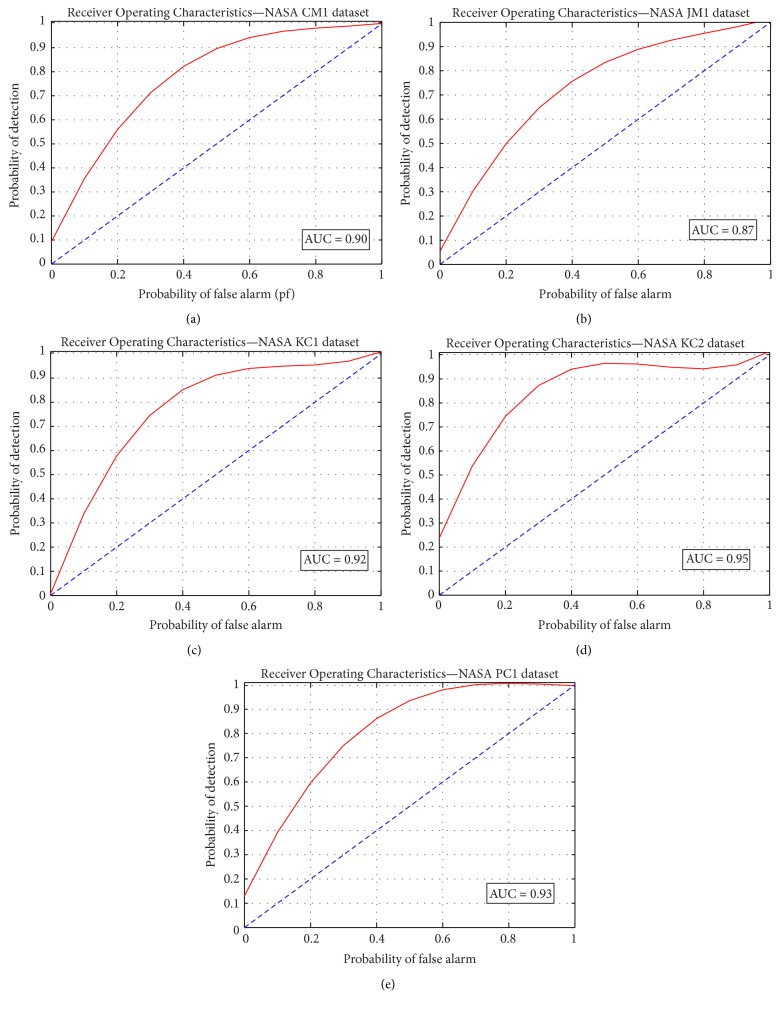
Receiver Operating Characteristics curve for five NASA datasets: (a) CM1, (b) JM1, (c) KC1, (d) KC2, and (e) PC1.

From Table 7, it can be noted that the area under curve value is noted to be greater than 0.5 and above 0.85, conveying that the proposed predictor model has resulted in acceptable solutions. With respect to accuracy and area under curve metrics, KC2 and PC1 datasets are observed to result in better solutions than the other three considered datasets. The proposed ADBBO-RBFNN without the cost factor is simulated for 30 trial runs and the specified solutions in Table 7 are obtained. The computed solutions in Table 7 prove the effectiveness and robustness of the non-cost-sensitive predictor model. Receiver Operating Characteristics are studied for the proposed classifier and the resulting plots are presented in Figures 5(a)–5(e). The ROC curve is generated for each execution of the cross-validation fold. ROC shows the grouping of good instances with that of the same class output.

Table 8 presents the comparison of the proposed classifier with the other algorithms applied for the same NASA datasets in terms of the performance metrics: sensitivity, specificity, probability of false alarm, balance, accuracy, area under curve, and error value. Results of Naïve Bayes, Random Forest, C4.5 Miner, Immunos, and ANN-ABC (Artificial Bee Colony) algorithm were considered from Arar and Ayan [5]; results of hybrid self-organizing map were taken from Abaei et al. [13]; and results of SVM, Majority Vote, and AntMiner+ were taken from Vandecruys et al. [14]. From Table 8, it is inferred that for the respective datasets the proposed adaptive dimensional BBO based non-cost-sensitive radial basis function neural network model is noted to produce better results with the earlier methods from the literature. It is to be noted that the solutions computed employing the traditional algorithms and that of the hybrid self-organizing maps follow semisupervised learning algorithmic procedures. With respect to AUC, the proposed ADBBO based RBFNN is noted to possess values nearer to 1, proving the validity of the results computed. The variation in accuracy of the proposed algorithm is noted to be high in comparison with the other classifiers, proving the effectiveness of the approach. The proposed predictor model seems to play well for KC1, KC2, and PC1 datasets better than for the CM1 and JM1 datasets.

### 6.2. Simulation Results for the Proposed Cost-Sensitive Prediction Model

The main focus made in this paper is the development of cost-sensitive radial basis function classifier model to classify the software entities that are defect-free or defect-prone. This subsection presents the computed solutions for the considered NASA datasets with cost-sensitive factor included as given in (21) for the proposed model.  Table 9 presents the results computed on employing the proposed classifier with four different cost ratios and their comparison of results with the existing methodologies from the literature [5]. The values of cost ratio are considered from the literature [5].

**Figure 6:**
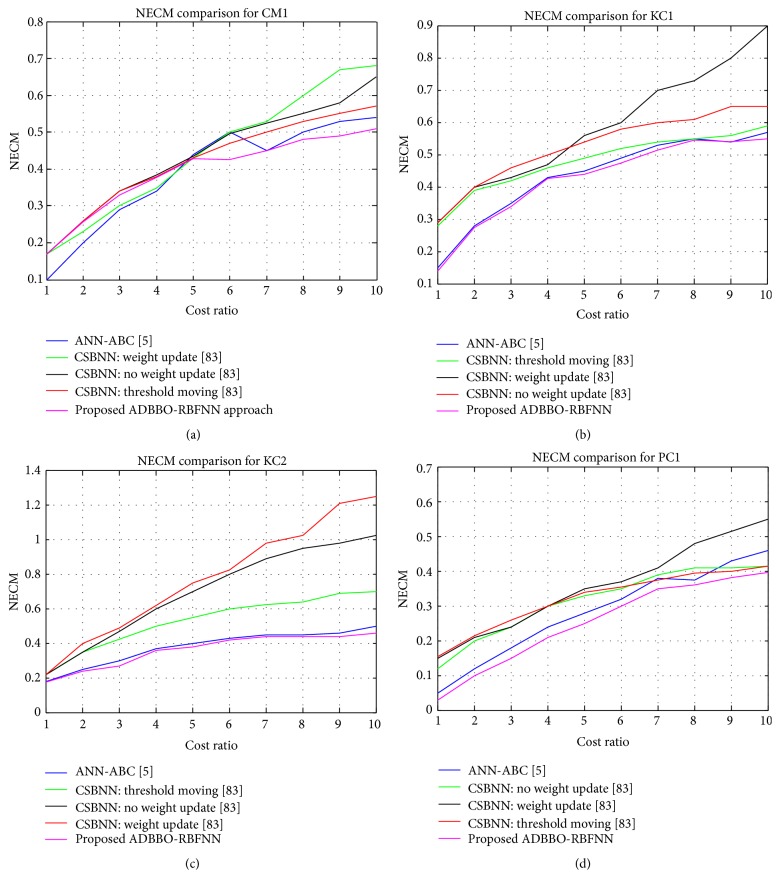
Cost ratio versus NECM comparison of the proposed model with other classifiers for NASA datasets (a) CM1, (b) KC1, (c) KC2, and (d) PC1.

From Table 9, it can be observed that when the cost ratio decreases the rate of probability of detection also decreases and this increases the probability of false alarm as well. Lower cost ratio results in higher accuracy rate. Also, lower cost ratio means minimal error in negative classes and, thus, this increases the accuracy rate. In comparison with the existing work [5], the proposed ADBBO based RBFNN classifier is noted to achieve better accuracy rate for the different cost ratios considered. This proves the effectiveness of the proposed model in detecting the defect-free and defect-prone developed software models. Further to the metrics probability of detection (pd), probability of false alarm (pf), and accuracy, the normalized expected cost of misclassification is also computed employing the proposed model. The convergence of the proposed algorithm is the minimization of this normalized expected cost of misclassification (NECM). Figures 6(a)–6(d) show the graphs computed for the NASA datasets with respect to cost ratio versus NECM.

In the proposed cost-sensitive model, NECM is employed as a key performance metric to analyze the prediction accuracy for the NASA datasets. The parameters of the algorithmic models are the same as that given in Table 6 for this cost-sensitive case also. The computed results employing the proposed ADBBO based cost-sensitive RBFNN model are compared to prove its effectiveness with the other existing classifiers from the literature: cost-sensitive boosting neural network [83] and cost-sensitive ANN-ABC model [5]. On comparing the NECM value computed, it is noted to be largely decreased with increased cost ratio factor with respect to other methods considered for comparison, proving its effectiveness. Cost-sensitive case is compared only for four datasets, as the literature results are not present for JM1 dataset. On performing the proposed algorithm for JM1 dataset, it is observed that the solution converges to a minimum of 0.44 when the cost ratio is 10. Thus, it is well noted that the proposed algorithmic predictor model has resulted in better solutions for the considered NASA datasets to predict defective models nearly for all cost ratios. It is well noted from Figure 6(a) that NECM value is minimal (as shown in pink color) as cost ratio increases in comparison with the other methods from the literature showing significant variation.

The novelty in this work includes the applicability of radial basis function (RBF) neuronal model for software detection. Earlier literatures reveal that this so-called RBF model has been applied for various fields like prediction, control, market analysis, image applications, and so on. This research paper applied this nonlinear neural network model for software defect analysis and optimized RBF neural network's weights which are of high importance using adaptive biogeography based optimization approach. The validation of the proposed approach is done with respect to the given comparison methods.

## 7. Conclusion

This paper proposed an adaptive dimensional biogeography based optimization based RBFNN classifier model to perform software defect prediction for the considered datasets from NASA PROMISE repository. Radial basis function neural network is a neuronal model employing Gaussian function to enable the network to attain fast convergence. In this paper, cost-sensitive RBFNN is developed along with a proposed variant of biogeography based optimization. The cost-sensitivity factor is added along with RBFNN to consider the effects of false positive and false negative costs. The results were simulated for both the non-cost-sensitive and the cost-sensitive case. The cost factors were noted to possess their influence on the probability of detection, probability of false alarm, and accuracy. The computed results of the proposed ADBBO-RBFNN predictor model are compared with the earlier existing algorithms in the literature on the five NASA datasets and the results obtained show that the performance is better for the proposed algorithm significantly.

## Figures and Tables

**Pseudocode 1:**
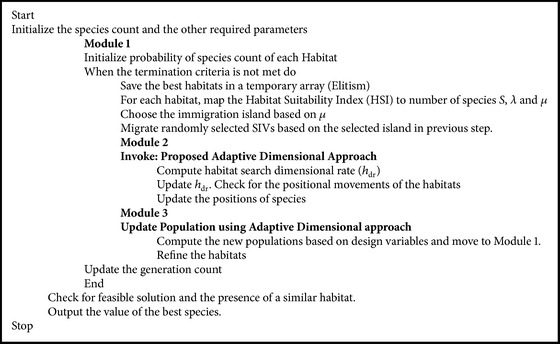
The proposed pseudocode of ADBBO algorithm.

**Pseudocode 2:**
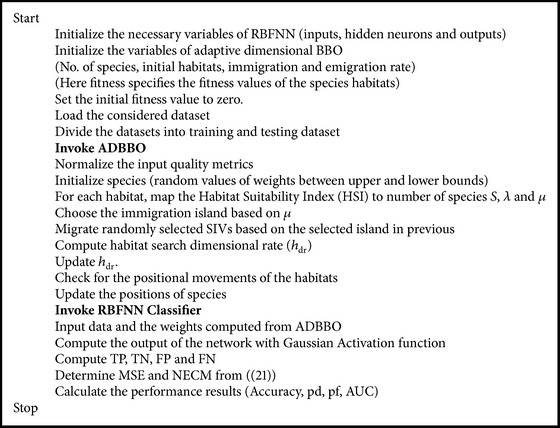
Pseudocode of the proposed ADBBO-RBFNN classifier.

**Table 1:** Description of datasets used in this study [12].

Name of the dataset	Dataset information	Language employed	Number of instances	Number of attributes	Nondefective module	Defective module	Defect rate
CM1	NASA spacecraft instrument	C	498	22	449	49	9.83%
JM1	Real-time predictive ground system	C	10885	22	8779	2106	19.35%
KC1	Storage management for receiving and processing ground data	C++	2109	22	1783	326	15.45%
KC2	Storage management for receiving and processing ground data	C++	522	22	415	107	20.49%
PC1	Flight software for Earth orbiting satellite	C	1109	22	1032	77	6.94%

**Table 2:** Attribute information of the datasets.

Number	Attribute type	Quality metrics	Attribute definition
1	McCabe's measure	loc	Line count of code
2		*v*(*g*)	Cyclomatic complexity
3		*ev*(*g*)	Essential complexity
4		*iv*(*g*)	Design complexity

5	Basic Halstead measures	loCode	Line count
6		loComment	Count of lines of comments
7		loBlank	Count of blank lines
8		loCodeAndComment	Count of code and comment lines
9		uniqOp	Unique operators
10		uniqOpnd	Unique operands
11		total_Op	Total operators
12		total_Opnd	Total operands
13		branchCount	Branch count of the flow graphs

14	Derived Halstead measures	*n*	Total operators + operands
15		*v*	Volume
16		*l*	Program length
17		*d*	Difficulty
18		*i*	Intelligence
19		*e*	Effort
20		*b*	Estimate of the effort
21		*t*	Time estimator

22	Output defect measure	Defects	{false, true}: module has/does not have one or more reported defects

**Table 3:** Selected attribute metrics to act as input for the proposed predictor model.

Sl. number	Name of the dataset	Number of attributes selected	Name of the selected attributes
1	CM1	7	loc, *iv*(*g*), *i*, loComment, loBlank, uniqOp, uniqOpnd
2	JM1	8	loc, *v*(*g*), *ev*(*g*), *iv*(*g*), *i*, loComment, loBlank, loCodeAndComment
3	KC1	8	*v*, *d*, *i*, loCode, loComment, loBlank, uniqOpnd, branchCount
4	KC2	3	*ev*(*g*), *b*, uniqOpnd
5	PC1	6	*v*(*g*), *i*, loComment, loCodeAndComment, loBlank, uniqOpnd

**Table 4:** Confusion matrix.

	Predicted as defective module	Predicted as nondefective module
Observed as defective module	True positive (TP)	False negative (FN)
Observed as nondefective module	False positive (FP)	True negative (TN)

True positive: correctly classified as defective module.

True negative: correctly classified as nondefective module.

False positive: classifies nondefective module as defective module.

False negative: classifies defective module as nondefective module.

**Table 5:** Performance measures.

Performance measures	Definition of the measure	Description
Sensitivity (or) Recall (or) True positive rate (or) Probability of detection (pd)	$\frac{TP}{TP+FN}$	Proportion of defective modules correctly predicted

Precision	$\frac{TP}{TP+FP}$	Proportion of modules predicted as defective

False positive rate (or) Probability of false alarm (pf)	$\frac{FP}{FP+TN}$	Proportion of nondefective modules predicted as defective

Specificity	$\frac{TN}{TN+FP}$	Proportion of correctly predicted nondefective modules

Classification accuracy	TN+TPTN+FN+FP+TPTN+TPXXTN+FN+FP+TPXX	Proportion of correctly predicted modules

Balance	$1-\frac{\sqrt{(0-pf{)}^{2}+(1-pd{)}^{2}}}{\sqrt{2}}$	Balance combines pf and pd into one measure and is defined as the distance from the ROC “sweet spot” (where pd* = *1 and pf* = *0)

Receiver Operating Characteristics (ROC) curve	A graphical plot of “pd” versus “pf” where the discrimination threshold is varied	

**Table 6:** Parameters of the proposed ADBBO-RBFNN algorithm.

Parameters	BBO	Parameters	RBFNN
Habitat size	40	Learning rate	0.2
Habitat modification probability	1	Momentum factor	0.1
Immigration probability bounds per gene	[0, 1]	Number of hidden neurons	1/2 the number of input neurons
Step size for numerical integration	1	Maximum iteration	500
Maximum immigration	1	Activation function	Gaussian activation function
Migration rate for each island	1	Number of output neurons	1 (defect or defect-free)
Mutation probability	0.005		
Maximum generation	500		
Habitat search dimensional rate *d*_*r*_	0.5		

**Table 7:** Performance results of the proposed predictor on the five NASA datasets (non-cost-sensitive case).

NASA datasets	Sensitivity (pd)	Specificity	False positive rate (FPR or pf)	Balance	Accuracy	Area under ROC (AUC)
CM1	81.92	80.96	29.71	75.41	82.57	0.90
JM1	79.85	82.31	36.22	70.69	77.03	0.87
KC1	85.67	87.95	20.24	82.46	84.96	0.92
KC2	87.96	86.24	17.93	84.73	88.65	0.95
PC1	77.84	89.33	30.23	73.49	86.29	0.93
Mean	82.65	85.36	26.87	77.36	83.90	0.9140

**Table 8:** Comparison results and error analysis on NASA datasets.

NASA datasets	Techniques	Sensitivity	Specificity	FPR or pf	Balance	Accuracy	AUC	MSE (error)
CM1	Naïve Bayes [5]	71.03	78.65	34.09	68.37	64.57	0.75	0.1456
	Random Forest [5]	70.09	71.29	32.17	68.94	60.98	0.74	0.2314
	C4.5 Miner [5]	74.91	74.66	27.68	73.58	66.71	0.53	0.3765
	Immunos [5]	73.65	75.02	30.99	71.24	66.03	0.63	0.1732
	ANN-ABC [5]	75.00	81.00	33.00	71.00	68.00	0.77	0.2435
	Hybrid self-organizing map [13]	70.12	78.96	30.65	69.73	72.37	0.80	0.0810
	Support vector machine [14]	78.97	79.08	31.27	73.35	78.69	0.79	0.0154
	Majority Vote [14]	79.80	80.00	30.46	74.16	77.01	0.81	0.1968
	AntMiner+ [14]	80.65	78.88	30.90	74.22	79.43	0.84	0.0345
	*Proposed ADBBO-RBFNN model*	*81.92*	*80.96*	*29.71*	*75.41*	*82.57*	*0.90*	*0.0067*

JM1	Naïve Bayes [5]	68.98	69.77	36.54	66.11	60.78	0.68	0.6547
	Random Forest [5]	66.72	72.38	33.47	66.62	63.97	0.75	0.6721
	C4.5 Miner [5]	69.08	68.55	40.67	63.87	62.35	0.61	0.5498
	Immunos [5]	70.99	70.21	43.00	63.32	64.55	0.63	0.4219
	ANN-ABC [5]	71.00	73.05	41.00	64.00	61.00	0.71	0.4057
	Hybrid self-organizing map [13]	71.02	74.90	40.57	64.75	72.33	0.82	0.5692
	Support vector machine [14]	70.89	79.00	39.87	65.09	70.32	0.81	0.3759
	Majority Vote [14]	74.65	73.46	40.36	66.30	75.92	0.83	0.0345
	AntMiner+ [14]	75.81	80.96	37.12	68.67	74.51	0.72	0.1786
	*Proposed ADBBO-RBFNN model*	*79.85*	*82.31*	*36.22*	*70.69*	*77.03*	*0.87*	*0.0156*

KC1	Naïve Bayes [5]	74.33	76.85	35.71	68.90	65.87	0.79	0.9854
	Random Forest [5]	72.54	75.89	37.91	66.90	67.99	0.80	0.6231
	C4.5 Miner [5]	76.42	75.64	34.05	70.71	68.01	0.64	0.7893
	Immunos [5]	78.05	72.91	36.92	69.63	63.55	0.71	0.6451
	ANN-ABC [5]	79.00	77.00	33.00	72.00	69.00	0.80	0.2257
	Hybrid self-organizing map [13]	80.92	80.94	35.67	71.40	78.43	0.86	0.1847
	Support vector machine [14]	81.37	81.27	28.96	75.65	79.24	0.83	0.5467
	Majority Vote [14]	82.65	85.62	30.98	74.89	79.66	0.85	0.0578
	AntMiner+ [14]	84.29	84.99	26.11	80.40	80.51	0.90	0.0346
	*Proposed ADBBO-RBFNN model*	*85.67*	*87.95*	*20.24*	*82.46*	*84.96*	*0.92*	*0.0239*

KC2	Naïve Bayes [5]	77.24	75.98	24.57	76.32	74.00	0.82	0.1453
	Random Forest [5]	70.32	70.71	23.90	73.05	77.81	0.82	0.5498
	C4.5 Miner [5]	69.87	74.67	29.19	70.34	76.54	0.67	0.6672
	Immunos [5]	76.51	75.92	25.06	75.71	72.90	0.73	0.4591
	ANN-ABC [5]	79.00	76.00	21.00	79.00	79.00	0.85	0.3195
	Hybrid self-organizing map [13]	80.98	77.82	23.09	78.85	85.98	0.91	0.1666
	Support vector machine [14]	84.35	78.96	25.61	78.78	87.12	0.88	0.2789
	Majority Vote [14]	86.71	84.77	20.38	82.80	83.47	0.82	0.1087
	AntMiner+ [14]	86.07	83.98	21.88	81.66	90.86	0.80	0.0985
	*Proposed ADBBO-RBFNN model*	*87.96*	*86.24*	*17.93*	*84.73*	*95.65*	*0.95*	*0.0067*

PC1	Naïve Bayes [5]	87.98	82.34	42.31	68.90	60.00	0.70	0.7689
	Random Forest [5]	82.31	80.99	46.71	64.68	63.98	0.85	0.6792
	C4.5 Miner [5]	76.58	81.76	38.24	68.29	62.18	0.68	0.5564
	Immunos [5]	81.99	79.66	39.00	69.62	61.73	0.64	0.4987
	ANN-ABC [5]	89.00	83.00	37.00	73.00	65.00	0.82	0.3125
	Hybrid self-organizing map [13]	86.79	85.67	35.60	73.15	95.87	0.87	0.1325
	Support vector machine (SVM) [14]	80.98	86.59	34.98	71.85	92.45	0.76	0.2037
	Majority Vote [14]	84.61	84.37	36.08	72.26	92.50	0.85	0.1078
	AntMiner+ [14]	89.34	87.12	37.29	72.58	91.85	0.91	0.0987
	*Proposed ADBBO-RBFNN model*	*90.89*	*89.33*	*30.23*	*73.49*	*96.29*	*0.93*	*0.0379*

**Table 9:** Performance and comparison results of the proposed predictor on the five NASA datasets (cost-sensitive case, four different cost ratios).

NASA datasets	Performance metrics	CR (cost ratio) = cost_false_negative_/cost_false_positive_							
		CR = 4.00		CR = 1.50		CR = 0.67		CR = 0.25	
		ANN-ABC [5]	Proposed model	ANN-ABC [5]	Proposed model	ANN-ABC [5]	Proposed model	ANN-ABC [5]	Proposed model
CM1	pd (or) TPR	91	**96**	75	**81**	62	**74**	21	**32**
	pf (or) FPR	60	**45**	38	**32**	28	**24**	5	**3**
	Accuracy	46	**61**	64	**72**	71	**79**	88	**93**

JM1	pd (or) TPR	99	**99**	80	**87**	44	**56**	8	**21**
	pf (or) FPR	94	**87**	54	**56**	17	**12**	2	**2**
	Accuracy	24	**35**	53	**76**	76	**88**	81	**97**

KC1	pd (or) TPR	93	**98**	87	**93**	59	**74**	22	**45**
	pf (or) FPR	58	**41**	44	**39**	20	**18**	4	**3**
	Accuracy	50	**76**	61	**84**	77	**90**	85	**97**

KC2	pd (or) TPR	90	**96**	80	**92**	74	**87**	37	**67**
	pf (or) FPR	39	**35**	27	**24**	19	**14**	5	**3**
	Accuracy	67	**78**	74	**86**	80	**94**	84	**97**

PC1	pd (or) TPR	94	**98**	89	**96**	66	**78**	23	**41**
	pf (or) FPR	48	**32**	36	**27**	23	**20**	2	**2**
	Accuracy	55	**62**	66	**79**	76	**87**	93	**95**
